# First-Principle Study of Rh-Doped Nitrogen Vacancy Boron Nitride Monolayer for Scavenging and Detecting SF_6_ Decomposition Products

**DOI:** 10.3390/polym13203507

**Published:** 2021-10-13

**Authors:** Zhen Shi, Sheng-Yuan Xia

**Affiliations:** 1 School of Electrical Engineering, Guangxi University, Nanning 530004, China; 2 State Key Laboratory of Power Transmission Equipment & System Security and New Technology, Chongqing University, Chongqing 400044, China; xiashengyuan@cqu.edu.cn

**Keywords:** high voltage equipment, dielectric material, sulfur hexafluoride (SF_6_), defect sensing and scavenging

## Abstract

The scavenging and detection of sulfur hexafluoride (SF_6_) decomposition products (SO_2_, H_2_S, SO_2_F_2_, SOF_2_) critically matters to the stable and safe operation of gas-insulated switchgear (GIS) equipment. In this paper, the Rh-doped nitrogen vacancy boron nitride monolayer (Rh-VNBN) is proposed as a gas scavenger and sensor for the above products. The computational processes are applied to investigate the configurations, adsorption and sensing processes, and electronic properties in the gas/Rh-VNBN systems based on the first-principle calculations. The binding energy (E_b_) of the Rh-VNBN reaches −8.437 eV, while the adsorption energy (E_ad_) and band gap (*BG*) indicate that Rh-VNBN exhibits outstanding adsorption and sensing capabilities. The density of state (DOS) analysis further explains the mechanisms of adsorption and sensing, demonstrating the potential use of Rh-VNBN in sensors and scavengers of SF_6_ decomposition products. This study is meaningful as it explores new gas scavengers and sensors of SF_6_ decomposition products to allow the operational status assessment of GIS equipment.

## 1. Introduction

Sulfur hexafluoride (SF_6_) is extensively applied in gas-insulated switchgear (GIS) equipment because of its good thermal conductivity, high dielectric strength, ideal arc-extinguishing properties, and chemical inertness [1,2,3]. Nevertheless, the long-term operation of GIS equipment inevitably results in latent insulation defects that cause partial discharges (PD) [4]. With the effect of PD, SF_6_ might decompose into SO_2_, H_2_S, SO_2_F_2_, and SOF_2_ [5,6,7]. The decomposition products would cause stronger discharges that would significantly reduce the insulation properties of the SF6 [8,9,10]; therefore, the detection of the SF6 decomposition products is necessary to ensure the reliability of GIS equipment [11,12].

Over the years, considerable attention has been dedicated to two-dimensional (2D) nanomaterials due to their excellent carrier mobility, high chemical activity, and high specific surface area [13,14,15]. General 2D nanomaterials such as graphene [16,17,18], boron nitride (BN) [19,20], and transition metal sulfides [21,22] are chemically sensitive materials with excellent performance, which have been researched extensively for gas sensing [23,24,25,26]; however, the selectivity is generally not satisfactory to obtain single 2D nanomaterial gas sensors. A variety of nanocomposite materials with a second phase, such as metals [27,28,29,30], metal oxides [31,32,33], and other materials, can be constructed through surface functionalization, thereby improving the sensitivity to certain specific gases. For example, the Rh-BN monolayer has been studied to produce workable SF_6_ decomposition gas sensors [34]. Meanwhile, 2D nanomaterials are defective for the most part [35]. The formed electronic variation regions between the defect and pristine material have significant impacts on the electronic and chemical properties of 2D nanomaterials [36,37,38]. Moreover, the nitrogen vacancy BN monolayer (VNBN) has better semiconducting properties and thermal stability [39,40,41]; however, the effects of the vacancy for BN on monitoring SF_6_ decomposition products are less well-understood.

In this study, the Rh-doped nitrogen vacancy BN monolayer (Rh-VNBN) is studied as a sensor and scavenger of the SF6 decomposition products SO_2_, H_2_S, SO_2_F_2_, and SOF_2_. The relevant theoretical calculations are based on first-principle density functional (DFT) theory. The adsorption and sensing processes of Rh-VNBN for SO_2_, H_2_S, SO_2_F_2_, and SOF_2_ are calculated and analyzed. The stable configurations of the gas/Rh-VNBN adsorption systems are presented. Moreover, the adsorption energy (E_ad_), band gap (*BG*), and electronic properties of these gas/Rh-VNBN adsorption systems are investigated. On this basis, this computational study first presents a gas scavenger and sensor based on Rh-VNBN, which features promising applicability for the scavenging and detection of SF_6_ decomposition products, thereby keeping GIS equipment safe and stable during operation.

## 2. Modeling of the Gas/Rh-VNBN System

### 2.1. Configurations of Rh-VNBN and Gas Molecules

**Figure 1:**
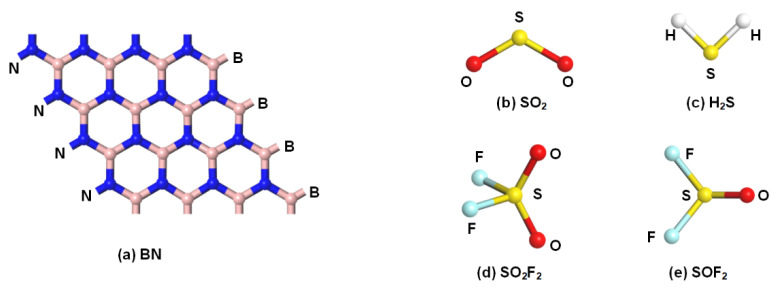
(**a**) Configurations of pure BN monolayer. (**b**–**e**) Configurations of the molecules of SF_6_ decomposition products.

Figure 1 displays the geometric configurations of pure BN monolayer and SF_6_ decomposition gases (SO_2_, H_2_S, SO_2_F_2_, SOF_2_). As can be seen in Figure 1a, BN exhibits a graphene-like two-dimensional hexagonal layered structure, which contributes to the increase in specific surface area. The bond length between the B atom and N atom is 1.47 Å. As shown in Figure 1b–e, unlike SO_2_ and H_2_S, SO_2_F_2_ and SOF_2_ have three-dimensional space structures. The high electronegativity and tetrahedral structure make the SO_2_F_2_ extremely stable, while the physicochemical properties of SOF_2_ are similar to SO_2_F_2_ [42,43,44].

**Figure 2:**
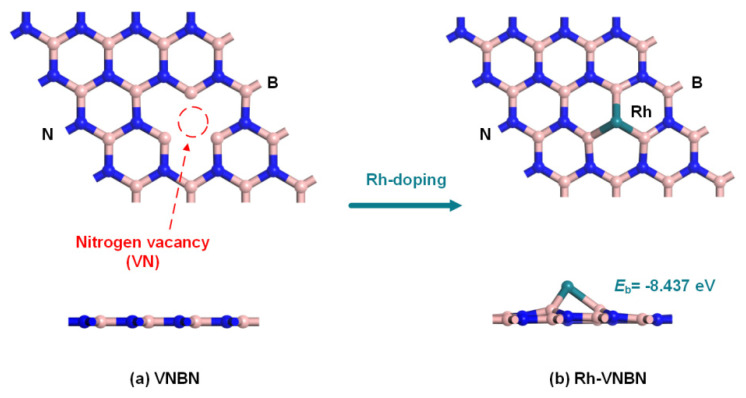
Configurations of the Rh-doped nitrogen vacancy BN monolayer. The red circle represents the nitrogen vacancy. (**a**) VNBN, (**b**) Rh-VNBN.

The configurations of VNBN and Rh-VNBN after geometric optimization are depicted in Figure 2a,b, respectively. The existence of the nitrogen vacancy (VN) creates additional energy states, as the VN has higher chemical stability as compared with the boron vacancy (VB) [27]. As can be seen in Figure 2b, the Rh atom occupies the VN and bonds to the three adjacent B atoms after doping and geometric optimization. From the front view, the Rh atom bonded to the three B atoms is slightly raised from the VNBN surface.

Generally, the binding energy (E_b_) of VNBN is negative after doping with the Rh atom, indicating that there is an exothermic doping process. This also suggests that the doping reaction occurs spontaneously; hence, the doped configurations of Rh-VNBN have higher stability with higher absolute binding energy values. As shown in Figure 2b, high E_b_ (−8.437 eV) suggests that the Rh atom is stably bonded to VNBN, forming a surface support and strong bonding force in the doping process [45,46,47]. The above calculation proves that there is a strong interaction between Rh and VNBN and that the Rh-VNBN configurations have high stability.

### 2.2. Electronic Properties of Rh-VNBN

**Figure 3:**
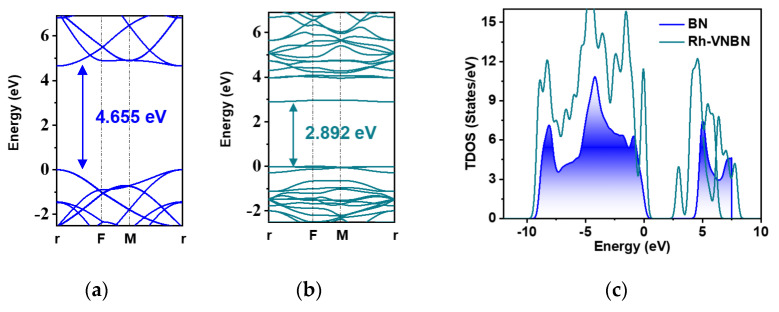

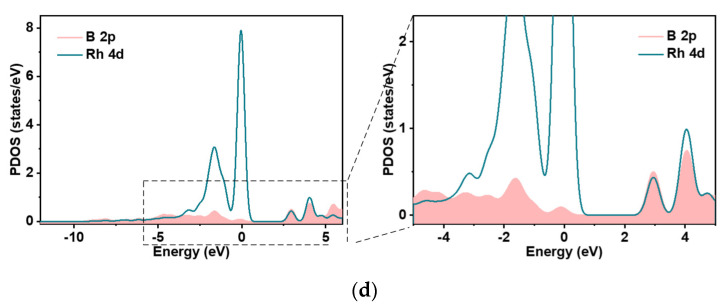
Electronic properties of Rh-VNBN: (**a**) band structure of pure BN; (**b**) band structure of Rh-VNBN; (**c**) TDOS analysis of BN vs. Rh-VNBN; (**d**) PDOS analysis of Rh-VNBN.

The band structures of the pure BN monolayer and Rh-VNBN are illustrated in Figure 3a,b, respectively. The figure indicates that the band gaps (*BG*) of the pure BN monolayer and Rh-VNBN are 4.655 eV and 2.892 eV, respectively. The lower band gap of Rh-VNBN indicates that the nitrogen vacancy and doped Rh atom will significantly increase the conductivity and improve the adsorption and sensing properties.

The total density of states (TDOS) and the partial density of states (PDOS) analyses were applied to describe the electronic properties of the nitrogen vacancy BN monolayer after doping with the Rh atom. As shown in Figure 3c, the TDOS of the pure BN monolayer shows notable semiconductor properties, with a band gap at the Fermi level. After doping with the Rh atoms into the nitrogen vacancy, some new states appeared in the TDOS of the Rh-VNBN at the Fermi level, indicating the nitrogen vacancy and the doped Rh atom contributes obviously to the TDOS. The change in TDOS is consistent with the change in BG, which means that electrons can be easily transferred from the valence band to the conduction band. It can be seen from the PDOS results in Figure 3d that the states of the B 2p orbitals highly overlap with those of the Rh 4d orbital within the range of −5 eV to 5 eV. The above electronic properties suggest strong bonding between the Rh atom and adjacent B atoms in the Rh-VNBN, leading to stable Rh-B bonds and a larger absolute binding energy.

### 2.3. Configurations of Gas/Rh-VNBN Adsorption Systems

**Figure 4:**
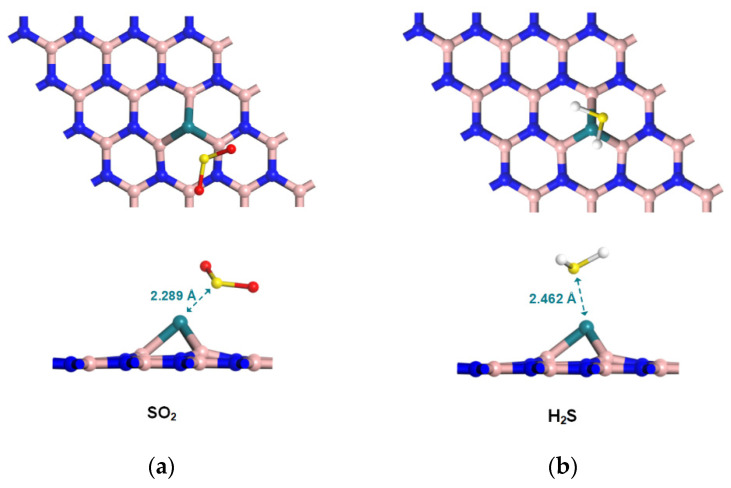

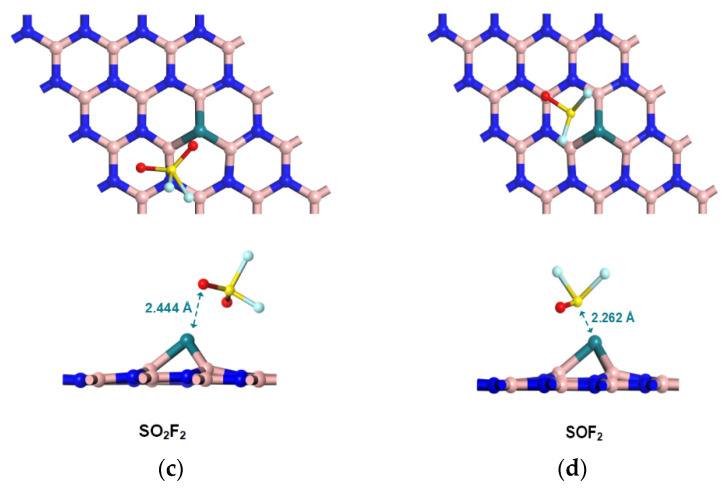
Configurations of gas adsorption systems: (**a**) SO_2_/Rh-VNBN adsorption system; (**b**) H_2_S/Rh-VNBN adsorption system; (**c**) SO_2_F_2_/Rh-VNBN adsorption system; (**d**) SOF_2_/Rh-VNBN adsorption system.

Different configurations of gas/Rh-VNBN pre-adsorption systems are considered in this computational study. Similar to binding energy, the adsorption energy (E_ad_) of the gas/Rh-VNBN system is generally negative, which corresponds to an exothermic, spontaneous gas adsorption process. With the higher absolute value of E_ad_, the SF_6_ decomposition gases are more easily adsorbed by the Rh-VNBN, which also results in higher stability of the gas/Rh-VNBN adsorption system. After comparing the adsorption energy, the stable systems of various SF_6_ decomposition products (SO_2_, H_2_S, SO_2_F_2_, SOF_2_) on Rh-VNBN are displayed in Figure 4. The adsorption distances between Rh-VNBN and various SF6 decomposition products (SO_2_, H_2_S, SO_2_F_2_, SOF_2_) are 2.289 Å, 2.462 Å, 2.262 Å, and 2.444 Å, respectively. Compared with the pre-adsorption systems, the distance between Rh-VNBN and the SF_6_ decomposition gases is shortened, which suggests a tendency of the SF_6_ decomposition gases to move toward the Rh-VNBN.

## 3. Results and Discussion

### 3.1. Adsorption and Sensing Properties of Gas/Rh-VNBN Adsorption System

**Figure 5:**
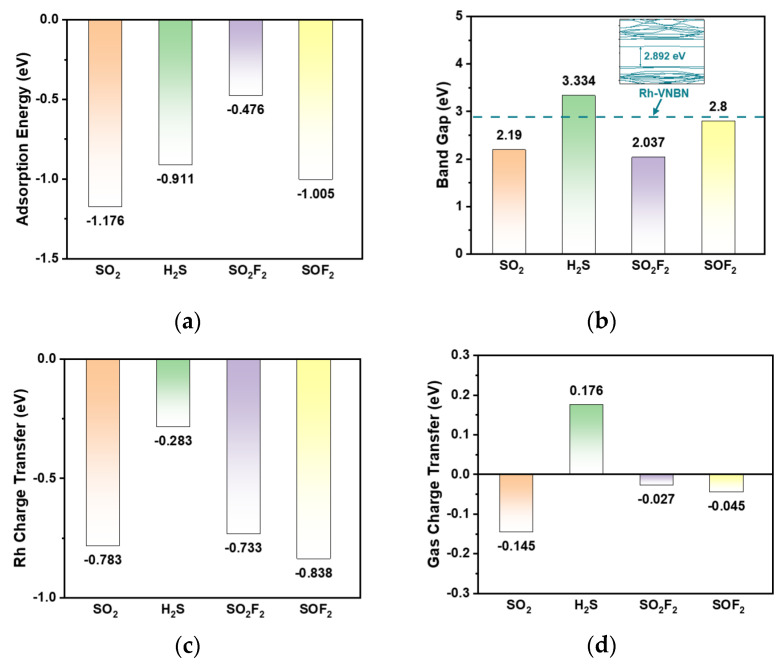
Adsorption and sensing properties of the gas/Rh-VNBN adsorption system: (**a**) E_ad_ values of the four gas/Rh-VNBN adsorption systems; (**b**) band gap values of the four gas/Rh-VNBN adsorption systems; (**c**) charge transfer values of the Rh atom in the four gas/Rh-VNBN adsorption systems; (**d**) charge transfer values of the gas molecules in the four gas/Rh-VNBN adsorption systems.

Figure 5a shows the adsorption energy values of the four gas/Rh-VNBN adsorption systems mentioned above. The adsorption energy values of SO_2_/Rh-VNBN, H_2_S/Rh-VNBN, SO_2_F_2_/Rh-VNBN, and SOF_2_/Rh-VNBN adsorption systems are −1.176 eV, −0.911 eV, −0.476 eV, and −1.005 eV, respectively. The adsorption energy of SO_2_F_2_/Rh-VNBN is lower than the other three adsorption systems and is physically absorbed by the Rh-VNBN. This indicates that the SO_2_F_2_/Rh-VNBN adsorption system is not as stable as the other three adsorption systems; however, it is important that the adsorption processes of SF6 decomposition products in Rh-VNBN are all spontaneous and stable.

As presented in Figure 5b, the BG values of SO_2_/Rh-VNBN, H_2_S/Rh-VNBN, SO_2_F_2_/Rh-VNBN, and SOF_2_/Rh-VNBN adsorption systems are 2.190 eV, 3.334 eV, 2.037 eV, and 2.800 eV, respectively. Moreover, the sensing analysis of the gas/Rh-VNBN adsorption systems is based on the changes of resistance. It is well known that the greater the change in BG, the greater the change in conductivity (σ) of the gas/Rh-VNBN system. As the resistance and σ are negatively correlated, the sensing properties of Rh-VNBN for SO_2_, H_2_S, and SO_2_F_2_ are consequently better than those for SOF_2_.

In addition, the Rh charge transfer (*Q*_Rh_) and Gas charge transfer (*Q*_gas_) based on the Mulliken population analysis are shown in Figure 5c,d, respectively. The charge transfer values of the Rh atom in the SO_2_/Rh-VNBN, H_2_S/Rh-VNBN, SO_2_F_2_/Rh-VNBN, and SOF_2_/Rh-VNBN adsorption systems are −0.783 eV, −0.283 eV, −0.733 eV, and −0.838 eV, respectively. The *Q*_Rh_ is negative in all four adsorption systems, indicating that Rh atom always loses electrons during the adsorption process. In Figure 5d, the charge transfer values of the gas molecules in the SO_2_/Rh-VNBN, H_2_S/Rh-VNBN, SO_2_F_2_/Rh-VNBN and SOF_2_/Rh-VNBN adsorption systems are −0.145 eV, 0.176 eV, −0.027 eV, and −0.045 eV, respectively. The *Q*_gas_ is negative for all adsorption systems except the SO_2_/Rh-VNBN adsorption system. This indicates that electrons are transferred from gases to Rh-VNBN during the adsorption process for most of the SF_6_ decomposition products (H_2_S, SO_2_F_2_, SOF_2_).

### 3.2. Electronic Properties and Mechanisms of the Gas/Rh-VNBN Adsorption Systems

**Figure 6:**
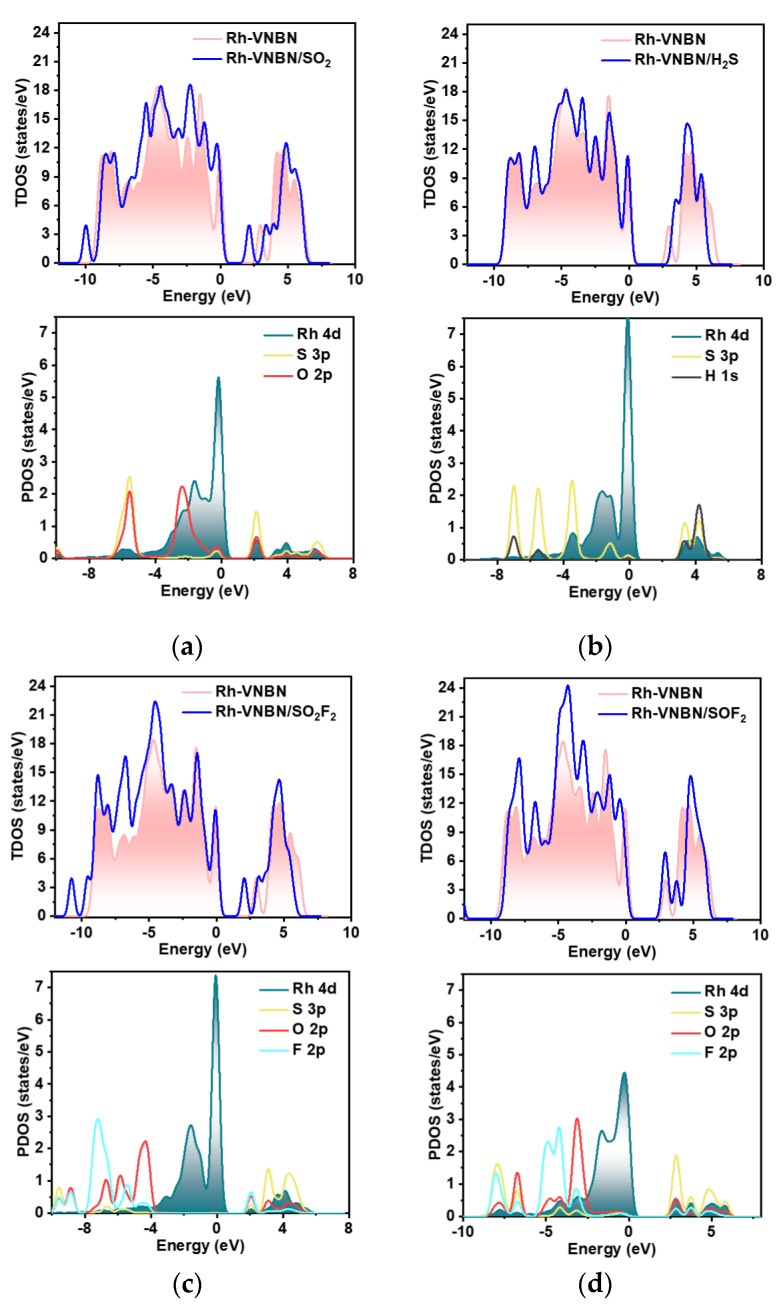
TDOS and PDOS analyses of gas/Rh-VNBN adsorption systems: (**a**) SO_2_/Rh-VNBN adsorption systems; (**b**) H_2_S/Rh-VNBN adsorption systems; (**c**) SO_2_F_2_/Rh-VNBN adsorption systems; (**d**) SOF_2_/Rh-VNBN adsorption systems.

The TDOS and PDOS analyses for various gas adsorption systems are used for in-depth investigation of the electronic properties of gas/Rh-VNBN adsorption systems to elucidate sensing and adsorption mechanisms. As presented in Figure 6, except for the H_2_S/Rh-VNBN adsorption system, the TDOS values near the Fermi level in the other adsorption systems shift slightly to the left, with more electrons appearing between the valence band and conduction band. As such, most of the adsorption is beneficial to the conductivity of the gas/Rh-VNBN systems; however, the conductivity of the H_2_S/Rh-VNBN adsorption systems decreases slightly, which is consistent with the change of the band gap.

From the perspective of PDOS, the Rh orbital in the SO_2_F_2_/Rh-VNBN adsorption system has relatively little overlap with the frontier atom orbitals of the gas molecule compared to the other three systems; therefore, the Rh 4d orbital is less hybridized with S 3p, O 2p, and F 2p of SO_2_F_2_. This suggests that the bonding force between Rh-VNBN and SO_2_F_2_ is not as strong as the other three gases (SO_2_, H_2_S, SOF_2_). The PDOS explains why the adsorption energy absolute value of SO_2_F_2_/Rh-VNBN is higher than the other three adsorption systems. Moreover, the Rh 4d orbital is strongly hybridized with frontier atom orbitals of SO_2_, H_2_S, and SOF_2_ gas molecules, demonstrating that the bonding force between Rh-VNBN and these three gases is strong. Correspondingly, the PDOS also illustrates that most gas/Rh-VNBN adsorption systems in this study are stable. In summary, the DOS values of gas/Rh-VNBN adsorption systems also further illustrate the potential of Rh-VNBN in sensors and scavengers of SF_6_-decomposed products.

## 4. Conclusions

In this study, Rh-VNBN is proposed for the scavenging and detection of SF_6_-decomposed products. First-principle calculations based on DFT theory are applied to study the configurations, adsorption and sensing process, and electronic properties of Rh-VNBN for SF_6_-decomposed products. The following conclusions are drawn from the present study.

The Rh atom occupies the nitrogen vacancy and bonds to the three adjacent B atoms to form a stable configuration of Rh-VNBN. The binding energy (E_b_) of Rh-VNBN reaches −8.437 eV, indicating that the Rh atom is stably bonded to VNBN. Moreover, the nitrogen vacancy and doped Rh atom significantly improve the conductivity of Rh-VNBN.

1. Based on the analysis of the adsorption energy (E_ad_), the adsorption processes of SF6 decomposition products on Rh-VNBN are spontaneous and stable. In addition, the changes of the band gap (BG) suggest the sensing properties of Rh-VNBN for SO_2_, H_2_S, and SO_2_F_2_ are better than that for SOF_2_. Rh-VNBN exhibits outstanding adsorption and sensing capabilities for various SF_6_-decomposed gases;
2. The electronic properties of gas/Rh-VNBN systems are studied, contributing to the understanding of the adsorption and sensing mechanisms. Additionally, the DOS further demonstrates the potential of Rh-VNBN for use in sensors and scavengers of SF6-decomposed products;
3. From a long-term perspective, this computational study on gas/Rh-VNBN adsorption systems is important for future research on scavengers and sensors of SF6-decomposed gases, thereby ensuring the safe and stable operation of GIS equipment.

## 5. Computational Details

All of the first-principle calculations were performed using the DMol^3^ package in Materials Studio (MS) based on DFT [48], which has been demonstrated to be reasonable in previous experimental and theoretical studies [15,49,50,51,52]. The electron exchange and correlation process was set to the generalized gradient approximation (GGA) of the Perdew–Burke–Ernzerhof (PBE) functional [53]. The double numerical basis with polarization (DNP) was used as the atomic orbital basis set, while the DFT semi-core pseudopotential (DSSP) was applied to process the relativistic effects of the Rh atom [54]. Considering the van der Waals forces of the gases and Rh-VNBN, the semi-empirical dispersion corrections (DFT-D) method proposed by Grimme was applied to better investigate long-range weak interactions [55]. The pure BN monolayer supercell, including 16 B and 16 N atoms, was built with a graphene-like structure. The k-point sample of the Monkhorst–Pack grid was sampled as 8 × 8 × 1 and 5 × 5 × 1 for electronic structure and geometry optimization calculations, respectively [56]. The convergence criteria adopted in this study, including the energy tolerance accuracy, maximum force, and maximum displacement, were set as 10^−5^ Ha, 2 × 10^−3^ Ha/Å, and 5 × 10^−3^ Å, respectively [57]. For static electronic structure calculations, a self-consistent loop energy of 10^−6^ Ha, global orbital cutoff radius of 5.0 Å, and smearing of 5 × 10^−3^ Ha were applied [30].

To identify the doping site of the nitrogen vacancy BN monolayer with the best stability, various Rh doping sites were considered and analyzed. In general, the binding energy (E_b_) of Rh-VNBN is adopted to assess the doping stability of the Rh atom. The E_b_ is determined using Formula (1) as presented below:

E_b_ = E_Rh-VNBN_ − E_Rh_ − E_VNBN_ (1)

where E_Rh-VNBN_, E_Rh_, and E_VNBN_ are the energy of the Rh-VNBN, Rh atom, and nitrogen vacancy BN monolayer, respectively.

Similarly, to identify the best stability of geometric configurations for SF_6_ decomposition gases (SO_2_, H_2_S, SO_2_F_2_, SOF_2_) adsorbed on the Rh-VNBN, the adsorption energy (E_ad_) of the gas/Rh-VNBN adsorption system is commonly applied to assess adsorption properties. The E_ad_ is calculated using the following formula:

E_ad_ = E_gas/Rh-VNBN_ − E_Rh-VNBN_ − E_gas_ (2)

where E_gas/Rh-VNBN_, E_Rh-VNBN_, and E_gas_ represent the total energy of the gas/Rh-VNBN adsorption system, isolated Rh-VNBN, and gas molecule, respectively.

Depending on the variation of the band gap (BG), the relevant conductivity (σ) changes of gas/Rh-VNBN system can be calculated using Formula (3):

σ ∝ exp(−BG/2kT)(3)

where T is the temperature in kelvin (K).
